# Legal Notes for Institutional Workers

**Published:** 1912-07-20

**Authors:** 


### LEGAL NOTES FOR INSTITUTIONAL WORKERS.

**(The Editor will be pleased to answer Legal queries in this column.)**

### Hysteria and the Workmen's Compensation Act.

To the cases illustrating certain "diseases," the onset of which has been said to be accidental, which have been enumerated in this column, that of "hysteria" has now to be added. In a case at Tunstall it appeared that in 1906 a man working in the Potteries was injured while wheeling a crab. He received compensation for incapacity to work right down to June 5 of the present year, when the employers sought to have the payments reduced on the ground that he had recovered sufficiently to be able to do some work. Mr. Carrington-Sykes, F.R.C.S., said the man was suffering from hysteria. He suggested that the cure was to take the man out of himself and away from his surroundings. He added: "I would stake my reputation that if a man in this condition had to earn his next meal before he got it, he could earn it." Dr. Shufflebotham said he could not imagine any work at a pottery that would suit the man. It seemed to him to be a very hopeless case. In giving judgment his Honour said: "In my view the man's condition has been a persistent state of mental injury." He dismissed the application, saying that the medical referee agreed with him.

### The Privilege of Medical Witnesses.

From time to time a protest is raised in this country on behalf of the medical practitioner who is called upon to disclose the secrets of the consulting room. Although this protest has never yet caught the sympathetic ear of the Legislature in England, some of our brethren in the Dominions succeeded many years ago in securing for the medical practitioner the same protection which we accord to a lawyer. Thus, by the Victoria Evidence Act, 1890, s. 55, it is provided that: "No physician or surgeon shall without the consent of his patient divulge in any civil suit, action, or proceedings (unless the sanity of the patient be the matter in dispute) any information which he may have acquired in attending the patient, and which was necessary to enable him to prescribe or act for the patient." We shall return to this subject again next week.

